# Pharmacological targeting of tau‐induced retrotransposon activation

**DOI:** 10.1002/alz70859_098658

**Published:** 2025-12-25

**Authors:** A. Campbell Sullivan, Gabrielle Zuniga, Paulino Ramirez, Roman A. Fernandez, Chen‐Pin Wang, Ji Li, Lisa Davila, Kristine Pelton, Sandra Gomez, Claira Sohn, Elias M. Gonzalez, Marisa Lopez‐Cruzan, David A. Gonzalez, Alicia S. Parker, Eduardo Marques Zilli, Gabriel A. de Erausquin, Sudha Seshadri, Sara Espinoza, Nicolas Musi, Bess Frost

**Affiliations:** ^1^ Glenn Biggs Institute for Alzheimer's & Neurodegenerative Diseases, University of Texas Health Science Center, San Antonio, TX USA; ^2^ Sam & Ann Barshop Institute for Longevity & Aging Studies, San Antonio, TX USA; ^3^ Center for Alzheimer's Disease Research, Providence, RI USA; ^4^ Glenn Biggs Institute for Alzheimer’s and Neurodegenerative Diseases, San Antonio, TX USA; ^5^ Glenn Biggs Institute for Alzheimer’s & Neurodegenerative Diseases, San Antonio, TX USA; ^6^ University of Texas Health San Antonio, San Antonio, TX USA; ^7^ Brown University Center for Alzheimer's Disease Research, Providence, RI USA; ^8^ Cedars‐Sinai Medical Center, Los Angeles, CA USA; ^9^ Barshop Institute for Longevity & Aging Studies, San Antonio, TX USA; ^10^ Rush University Medical Center, Chicago, IL USA

## Abstract

**Background:**

Approximately 40% of the human genome is composed of retrotransposons, virus‐like sequences that are typically silenced at the transcriptional and post‐transcriptional levels. We have previously discovered that retrotransposons are activated in a variety of human neurodegenerative disorders, including Alzheimer’s disease and related tauopathies, and causally drive neurotoxicity. An accumulating body of literature suggests a beneficial effect of targeting retrotransposon activation with 3TC, a nucleoside analog reverse transcriptase inhibitor, in the context of tau pathology.

**Method:**

To investigate the feasibility and safety of targeting retrotransposon activation in Alzheimer's disease, a phase 2a open‐label trial (NCT04552795) was conducted with 12 participants diagnosed with cognitive impairment due to suspected Alzheimer’s disease (MMSE > 24, CDR = 0.5). Participants were treated daily with 300 mg of 3TC for 24 weeks. 75% of the study population were women; 25% were from underrepresented groups. Additional objectives were to assess target engagement and gain preliminary insight into the effects of 3TC on cognition and fluid‐based biomarkers of neurodegeneration and neuroinflammation.

**Result:**

The treatment was well‐tolerated, with no significant safety concerns. Cognitive function remained stable; significant changes were observed in fluid biomarkers. Notably, glial fibrillary acidic protein (GFAP) levels in cerebrospinal fluid decreased significantly (*P* = 0.03), suggestive of reduced neuroinflammation. Plasma levels of Aβ42/40 were elevated (*P* = 0.009), suggestive of reduced amyloid plaque burden in the brain.

**Conclusion:**

The findings from this pilot study support the safety and potential efficacy of 3TC in targeting retrotransposon activation in Alzheimer's disease. The potential reduction in neuroinflammation and amyloid plaque burden warrants further investigation in a larger, placebo‐controlled trial to confirm these results and explore the clinical relevance of our approach.

